# The Titanic Disaster

**Published:** 1912-05

**Authors:** 


### The Titanic Disaster.

So much has been said of the terrible disaster that we shall try, difficult as may be, to limit ourselves to a cold-blooded consideration of certain phases not too remote from medical science. In the February issue, referring to the Equitable Building fire, we suggested that a study of caloric values, quite in analogy to that of the dietetician, might have a practical bearing in establishing quantitative though approximate estimates of the amount of combustible material that might safely be allowed in the structure and contents of buildings. In the present connection, we would suggest as a text to the officers of steamship companies: $M = v\ w$. Momentum equals the product of velocity and weight. It ought to be possible to establish pretty acccurately, the resistance of various parts of a ship. Knowing w, the weight or displacement of the ship, it should be possible to establish a maximum value for v, the velocity, in case of danger of collision either with another ship, an iceberg or a rock.

Expert sailors declare that the average well built ship of say 10,000 to 20,000 tons, can outride any storm, if there is plenty of sea room; that with reasonable caution as to lights and whistles, there is practically no danger of collision, even in a fog, with another vessel, provided of course that both vessels are carefully conducted; that, unless prevented from making observations, or driven by a storm, there is no danger of striking a rock; but that the danger of icebergs remains paramount.

Four years ago, but almost a month later in the season, and in a milder season, at that, we had some opportunity to note how a careful captain would solve the above equation for v. For two entire days, we averaged about seven miles an hour, never exceeding ten, and sometimes merely floating when more expert eyes than ours could differentiate ice from fog. This was for a vessel of about a quarter of the tonnage of the Titanic. The mere fact that the enormous dimensions of the Titanic do not show a tonnage displacement corresponding to the cube of similar diameters, indicates that bigness and strength are not synonymous. If the resistance of individual parts were equal on the small and the big ship, it is obvious that the latter should, with equal caution, have proceeded at a rate of less than two miles an hour, and that, in any event, a very slow speed was required, quite ignoring the important fact that a small ship can be handled and stopped much more quickly than a large one. In advance of definite testimony, it was assumed that the Titanic was making about 15 miles an hour, and struck head-on with a momentum graphically compared to that of 37 Empire State express trains at 70 miles an hour. It was asserted—and readily conceded by non-expert readers,—that this momentum would shear off bolts and otherwise disrupt the water-tight compartments throughout. The fact seems to be that the speed was 20—23 knots (approximately 25 land miles) an hour and that the ice was seen just in time to allow a rapid turn which caused practically the whole side of the ship to be torn, and that this fatal traumatism caused an almost imperceptible shock. As an instance of the difficulty of passing judgment in technical matters, it is now stated that, while the officer on the bridge acted properly in making the turn, if a head-on collision had been allowed, the ship would probably have floated.

The discussion of physical forces may seem academic, but it has a practical side, which should be legally established, on the mathematic basis.

We do not intend to throw blame on the captain of the Titanic. The mere fact that this, and other ships were following the northern route; that the presence of icebergs was well known, that the Titanic herself had transmitted a report of their observation only about one hour's average run from her position at the time, and that she struck about two hours afterward, shows that a tangible risk was being run, which no sane man would have incurred without orders. We must consider something more than the momentum of velocity and weight. It has been estimated that a few prominent passengers on the Titanic, represented nearly 200 million dollars; that five to ten million dollars' worth of jewelry were worn, or ready to be worn, on the ship; many of the passengers were of weight aside from money and social standing. It was the initial trip of the greatest boat that had ever been built. The whole civilized world was interested. While not built to rival the recent speed records, a schedule was expected about as short as the shortest even five years ago. The manager of the line was on board. This was the momentum. It struck a human being, a captain of long experience, who had never had, scarcely ever seen a marine disaster. He was careful, conscientious, in supreme authority, until he reached land. He could have taken the southern course, he could have kept the ship barely moving for several days. And then, even before his arrival, thanks to wireless, he would have been the employee, who had disgraecd a great company, who had shown the white feather, who had offended that part of the community which, on account of its wealth, demands promptness, obedience, good service. That was the object on which the momentum was expended.

The result of the physical and psychic momentum was a mortality, in a space of at most four hours, approximately equivalent to that for a whole year, of a city of 100,000 inhabitants—*Albany for example. This was a preventable mortality, preventable by the simple means of working out comparatively simple problems in elementary physics.

By the way, will this mortality show in the vital statistics? If so, where? If not, should it? If these—and many similar, scattered deaths remain unaccounted for in our tables, how will they affect subsequent statistics?

The necessity of an adeqate supply of life boats might seem to require no comment but one point in this connection seems not to have occurred to the multitude of writers on the subject. Up to recently there has been a growing opinion—which as a passenger we confess to have shared—that a life boat was either entirely useless or afforded a very doubtful chance of saving life and was rather a means of prolonging the inevitable suffering of a wreck. The prevalent ignorance of swimming by deep sea sailors is another expression of the same thought. With the development of wireless telegraphy, and the increase of risk inherent to the ship, such as dangerous speed, explosion of boilers, dropping of heavy engines through the bottom, etc., the value of the life boat again becomes worth the attention of business interests and of the law.

*Actually the death rate of Albany is much higher than the average.

